# Appendiceal Mixed Adeno-Neuroendocrine Carcinoma: A Population-Based Study of the Surveillance, Epidemiology, and End Results Registry

**DOI:** 10.3389/fonc.2016.00148

**Published:** 2016-06-13

**Authors:** Shayna Brathwaite, Martha M. Yearsley, Tanios Bekaii-Saab, Lai Wei, Carl R. Schmidt, Mary E. Dillhoff, Wendy L. Frankel, John L. Hays, Christina Wu, Sherif Abdel-Misih

**Affiliations:** ^1^Department of General Surgery, The Ohio State University Wexner Medical Center, Columbus, OH, USA; ^2^Department of Pathology, The Ohio State University Wexner Medical Center, Columbus, OH, USA; ^3^Department of Internal Medicine, The Ohio State University Wexner Medical Center, Columbus, OH, USA; ^4^Center for Biostatistics, The Ohio State University Wexner Medical Center, Columbus, OH, USA; ^5^Division of Surgical Oncology, The Ohio State University Wexner Medical Center, Columbus, OH, USA

**Keywords:** mixed adeno-neuroendocrine carcinoma, colonic neoplasms, appendiceal neoplasm, SEER

## Abstract

**Introduction:**

Mixed adeno-neuroendocrine carcinoma (MANEC) is a rare pathological diagnosis recently defined by the World Health Organization (WHO) in 2010. Prior to the definition by the WHO, tumors with both adenocarcinoma and neuroendocrine components were given multiple pathological designations making it difficult to characterize the disease. The aim of our study is to better characterize MANEC to better understand its natural history to influence patient care and positively impact outcomes.

**Materials and methods:**

The surveillance, epidemiology, and end results program database was queried for all patients aged 18 years or older between 1973 and 2012 who had the diagnosis composite carcinoid (*n* = 249) of the appendix. Composite carcinoid tumors refer to tumors that have both adenocarcinoma and carcinoid tumor components present, consistent with that pathological diagnosis MANEC. For comparison, the database was also queried for carcinoid tumor of the appendix (*n* = 950), signet ring cell carcinoma of the appendix (*n* = 579), and goblet cell carcinoid (GCC) tumors of the appendix (*n* = 944). The data were retrospectively reviewed, and clinicopathological characteristics, treatment regimens, and survival data were obtained.

**Results:**

The median age of diagnosis of MANEC tumors was 58 years of age. Eighty percent of patients were White, and 49% were female. Fifty-four percent of patients underwent hemicolectomy and 31% had partial/subtotal colectomy as their surgical management. Median overall survival for MANEC was 6.5 years (95% CI 4.5–9.7), which was statistically significantly shorter (*p* < 0.0001) in comparison to 13.8 years (95% CI 12.1–16.5) for GCC, 2.1 years (95% CI 1.8–2.3) for signet ring cell carcinoma, and 39.4 years (95% CI 37.1–NA) for carcinoid tumors.

**Discussion:**

MANEC is a more aggressive clinical entity than both GCC of the appendix and carcinoid tumors of the appendix. Based on these findings, patients with MANEC tumors should undergo aggressive multidisciplinary cancer management.

## Introduction

Mixed adeno-neuroendocrine carcinoma (MANEC) is a rare pathological diagnosis recently defined by the World Health Organization (WHO) in 2010 (1). Tumors are given the designation MANEC when they have both epithelial and neuroendocrine components, and each represents at least 30% of the tumor (1). Additionally, two out of the three commonly used pathological neuroendocrine markers, synaptophysin, chromogranin, and CD56, must be present (1, 2). Data are lacking regarding staging and prognosis for this histological subtype.

Prior to the definition by the WHO, tumors with both epithelial and neuroendocrine components were given multiple pathological designations making it difficult to characterize the disease. Designations included but are not limited to, goblet cell carcinoid (GCC), adenocarcinoma ex GCC, composite tumors, adenocarcinoid, collision tumors, and mixed endocrine–exocrine tumors (3–6). MANEC tumors have been found in the colon, rectum, appendix, stomach, and biliary tract, but reports primarily consist of case reports and small series (7–10). In particular, primary tumors of the appendix are very rare and only found in approximately 0.9–1.4% of appendectomy specimens (11, 12). The histological subtype of the appendiceal tumor plays a significant role in the biological behavior of the tumor and the overall survival (OS). The frequency of different histological subtypes varies, 11–20% are carcinoid, 14–19% GCC, 25–27% adenocarcinoma, 37% mucinous adenocarcinoma, and 4–6% signet ring cell carcinoma (13, 14). Due to the recent pathological designation by the WHO, it is likely that some of the subtypes presented above may include MANEC tumors. Therefore, the true incidence and biological behavior of the disease are largely unknown.

The majority of published literature about MANEC includes small case series and case reports; therefore, there is limited data regarding the clinical behavior of MANEC tumors. It is currently unclear whether MANEC is more biologically similar to its carcinoid/neuroendocrine or adenocarcinoma components (10, 15, 16). Classification as appendiceal carcinoid or neuroendocrine tumor is associated with a more indolent biology and often requiring only appendectomy for treatment. On the other hand, classification as an adenocarcinoma often requires more aggressive management with consideration for right hemicolectomy and systemic therapy depending on pathological disease staging. With recent recognition of MANEC as a distinct clinical entity, understanding the natural history and prognosis of MANEC is necessary to ensure proper and optimal management of these patients.

The aim of our study is to use the national surveillance, epidemiology, and end results (SEER) database to better characterize MANEC to understand the clinical behavior and biology of this disease.

## Materials and Methods

Protocol approval was obtained from the Ohio State University (OSU) Wexner Medical Center Institutional Review Board to query the SEER Program of the National Cancer Institute (NCI) database. The SEER database contains cancer incidence and survival data for population-based cancer registries that cover approximately 30% of the United States population (17). Data are included for patients aged 18 years or older between 1973 and 2012 (18). The database was queried using *International Classification of Diseases for Oncology 3* (ICD-O-3) histology and behavior codes. The histological subtypes were classified as follows: composite carcinoid of the colon and rectum (8244/3), GCC of the appendix (8243), signet ring cell carcinoma of the appendix (8490), and carcinoid/neuroendocrine tumor of the appendix (8240). Composite carcinoid tumors refer to tumors that have both adenocarcinoma and carcinoid tumor components present where at least 30% of each subtype is present in the tumor, consistent with WHO classification for MANEC. As MANEC is a fairly new pathological designation, the SEER database has not been updated with this designation, but the definition of composite carcinoid is consistent with MANEC, so it was used for the purposes of this study.

There were 431 patients identified with composite carcinoid of the colon and rectum (SEER MANEC equivalent), 950 carcinoid tumor of the appendix, 579 signet ring cell carcinoma of the appendix, and 944 GCC tumors of the appendix. The majority of MANEC tumors arose in the appendix (58%) with the remaining tumors arising in the cecum (15%), rectum (9%), descending colon (1%), sigmoid colon (4%), and ascending colon (4%). With the non-MANEC histologies being appendiceal only, the appendiceal MANEC (*n* = 249) were used for clinicopathological and survival comparisons.

The data were retrospectively reviewed, and demographics, clinicopathological characteristics, treatment regimens, and survival data were obtained.

### Statistical Analysis

Demographics and clinical characteristics were summarized using descriptive statistics. Chi-square test was used to compare categorical variables and Kruskal–Wallis test was used to compare age and tumor size. OS was calculated from the date of diagnosis to death from any cause. Survival curves were estimated using the method of Kaplan–Meier. Survival curves were compared between groups using log-rank test. Bonferroni method was used to adjust for multiple comparisons. In order to control for the effect of age and stage, Cox proportional regression model was also used to examine the association between OS and the disease groups. Estimated median with 95% confidence intervals were provided. *p* Values <0.05 were considered statistically significant. All statistical analyses were conducted using SAS version 9.3 (SAS Institute, Cary, NC, USA).

## Results

The demographic data of the patients with MANEC, GCC, signet ring carcinoma, and carcinoid/neuroendocrine carcinoma that were identified were compared and summarized in Table 1. The median age of diagnosis of MANEC (composite carcinoid) tumors was 58 years of age, which was comparable to signet ring carcinoma (median 58 years) and GCC (median 56 years), but was significantly older in comparison to carcinoid (median 40 years) (*p* < 0.0001). Eighty percent of MANEC patients were White, which was comparable to GCC (84%), signet ring cell carcinoma (79%), and carcinoid tumors (81%). There was a fairly equal gender distribution with 49% females and 51% males.

**Table 1:** **Patient demographics**.

	MANEC (*n* = 249)	Signet ring (*n* = 579)	GCC (*n* = 944)	Carcinoid (*n* = 950)	*p*-Value
Age, median (range)	58 (10–86)	58 (25–90)	56 (18–99)	40 (9–89)	<0.0001*
Gender, no. (%)					<0.0001*
Female	122 (49)	346 (60)	460 (49)	630 (66)	
Male	127 (51)	233 (40)	484 (51)	320 (34)	
Race, no. (%)					<0.0001*
White	199 (80)	456 (79)	790 (84)	767 (81)	
Black	21 (8)	52 (9)	78 (8)	67 (7)	
Asian/Pacific Islander	4 (2)	30 (5)	32 (3)	23 (2)	
Hispanic	22 (9)	38 (7)	37 (4)	76 (8)	
American Indian/Alaska Native	1 (<1)	2 (<1)	1 (<1)	3 (<1)	
Unknown	2 (<1)	1 (<1)	6 (<1)	14 (2)	

*Statistical significant *p*-value <0.05.

The clinicopathological characteristics of the different histologies were compared, as illustrated in Table 2. Pathologically, 24% of MANEC tumors were poorly differentiated, which was a statistically significant higher proportion in comparison to GCC (5%) (*p* < 0.0001). Additionally, MANEC tumors were more likely to have distant spread at the time of diagnosis (31%), in comparison to GCC (9%) and carcinoid (9%) (*p* < 0.0001). Notably, 59% of signet ring cell carcinoma present with distant disease.

**Table 2:** **Clinicopathological characteristics**.

	MANEC (*n* = 249)	Signet ring (*n* = 579)	GCC (*n* = 944)	Carcinoid (*n* = 950)	*p*-Value
Grade, no. (%)					<0.0001*
I. Well differentiated	22 (9)	18 (1)	70 (7)	174 (18)	
II. Moderately differentiated	13 (12)	36 (6)	61 (7)	40 (4)	
III. Poorly differentiated	59 (24)	297 (51)	47 (5)	7 (<1)	
IV. Undifferentiated, anaplastic	9 (4)	30 (5)	2 (<1)	4 (<1)	
Unknown	128 (51)	208 (36)	764 (81)	725 (76)	
Stage, no. (%)					<0.0001*
I	19 (8)	27 (5)	105 (22)	212 (52)	
II	107 (45)	106 (20)	302 (63)	73 (18)	
III	39 (16)	90 (17)	34 (7)	84 (20)	
IV	73 (30)	317 (59)	42 (9)	31 (8)	
Median overall survival (years)	6.5	2.1	13.8	39.4	<0.0001*

*Statistical significant *p*-value <0.05.

For MANEC patients, 8% were diagnosed with Stage I disease, 45% with Stage II, 16% with Stage III, and 30% with Stage IV, which was significant in comparison to GCC, signet ring, and carcinoid tumors (*p* < 0.0001) (Table 2). In examining stage IV disease at presentation, MANEC presented with 30% compared with 59, 9, and 8% for signet ring, GCC, and carcinoid, respectively.

**Figure 1:**
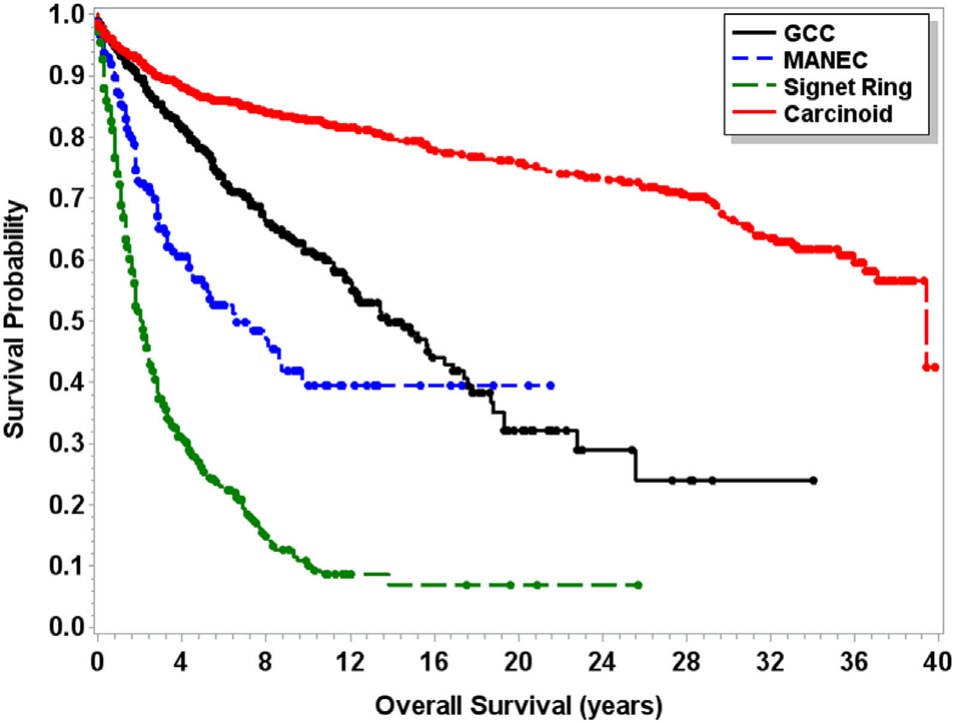
**Kaplan–Meier survival curve for patients with carcinoid tumors, GCC, MANEC, and signet ring cell carcinoma**.

**Figure 2:**
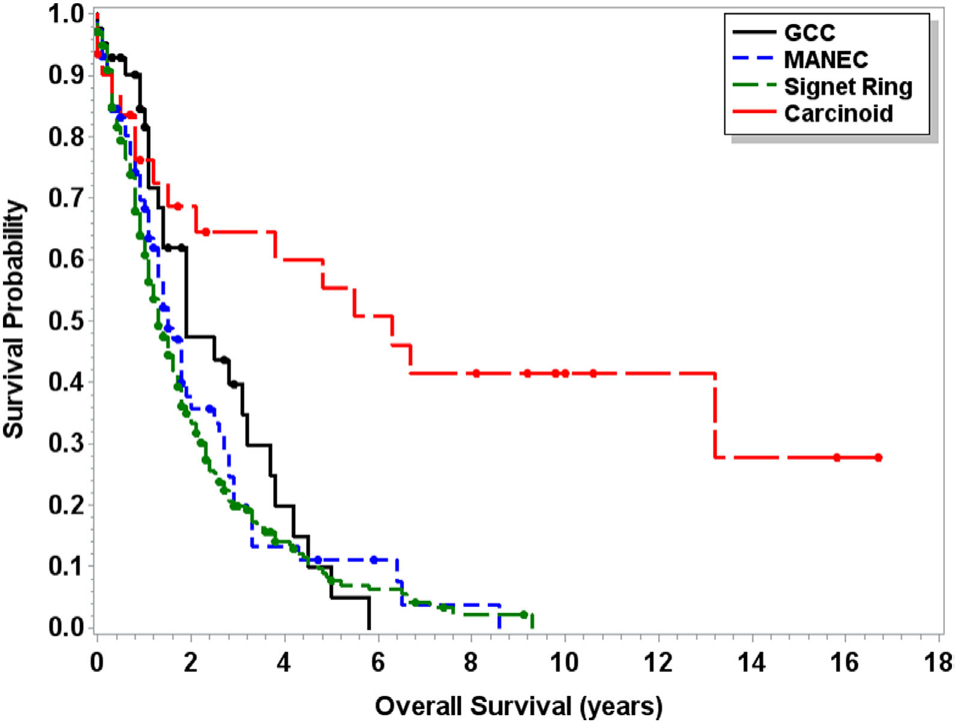
**Kaplan–Meier survival curve for patients with Stage IV only carcinoid tumors, GCC, MANEC, and signet ring cell carcinoma**.

Median OS for MANEC was 6.5 years (95% CI 4.5–9.7), which was statistically significantly shorter (*p* < 0.0001) in comparison to both 13.8 years (95% CI 12.1–16.5) for GCC and 39.4 years (95% CI 37.1–NA) for carcinoid tumors but longer in comparison to 2 years (95% CI 1.8–2.3) for signet ring cell carcinoma (Figure 1). Subgroup analysis was performed for all patients with “Colon excluding rectum” as their cause of death, to account for disease-specific survival. Median disease-specific survival for MANEC was 1.5 years (95% CI 1.1–1.8), which was similar to signet ring 1.3 years (95% CI 1.2–1.5) and carcinoid 1.8 years (95% CI 0.8–3.8), but statistically significantly shorter in comparison to 2.3 years (95% CI 1.7–2.8, *p* = 0.007) for GCC. Importantly, the OS for Stage IV MANEC was 1.5 years (95% CI 1.2–1.9), which was shorter than carcinoid (6.3 years, 95% CI 1.5–NA, *p* = 0.005). OS for Stage IV MANEC was similar to that of signet ring (1.3 years, 95% CI 1.2–1.6) and GCC (1.9 years, 95% CI 1.3–3.2) (Figure 2). Over time, the classification of tumors with both neuroendocrine and adenocarcinoma has changed. MANEC was recognized as distinct entity by SEER in 2007 and then was further defined by the WHO in 2010. OS for patients diagnosed in or after 2007 for MANEC was not reached (NR) (95% CI 3.7–NR) and for signet ring cell carcinoma was 2.2 years (95% CI 1.8–2.7). For GCC and carcinoid tumor, median OS was NR. As the distribution of stage varies among tumor types and there are differing ages at diagnosis, cox proportional regression model was used to assess association between OS and cancer type, controlling for age at diagnosis and stage. There is a statistically significant increased hazard from MANEC in comparison to carcinoid tumor [hazard ratio (HR) 3.3 (95% CI 2.8–4.9)] (*p* < 0.0006). There is no significant difference in hazard between GCC and MANEC [HR 0.829 (95% CI 0.6–1.1) (*p* = 1)]. Fifty-four percent of MANEC patients underwent hemicolectomy, and 31% had partial/subtotal colectomy as their surgical management.

## Discussion

Carcinoid/neuroendocrine tumors are typically considered to be less aggressive tumors as 1- and 5-year survival rates have been reported to be as high as 98.1 and 88.7%, respectively. Some studies have shown 10-year survival rates as high as 91% even in patients with positive lymph nodes (19–21). Consistent with our findings, GCCs of the appendix have been regarded as more aggressive than carcinoid/neuroendocrine tumors with regard to the extent of disease spread at diagnosis and the number of cases of lymph node involvement (14). Signet ring cell carcinoma represents the most aggressive histology with 76% of patients presenting with metastatic disease (14).

Due to the recent pathological designation of MANEC, there is little long-term outcome data and literature to guide clinical management. Prior to its definition as a distinct clinical entity, MANEC was often treated similar to their less aggressive counterparts, GCCs and carcinoid/neuroendocrine tumors of the appendix. This analysis of the SEER database represents the largest retrospective analysis of MANEC patients. Based on our analysis of the SEER database, MANEC appears to represent a more aggressive histology than both carcinoid tumors and GCC. We found OS for MANEC of 6.5 years (95% CI 4.5–9.7) to be significantly shorter than that of both carcinoid tumors and GCC. Additionally, a large proportion of patients were diagnosed with Stage IV disease (30%) in comparison to only 8% of carcinoid tumors and 9% of GCC. Furthermore, carcinoid tumors have a significantly lower age at diagnoses of 40, in comparison to 58 for MANEC and signet ring and 56 for GCC. The lower age at diagnosis and variations in proportions of stage may act as confounders in our survival analysis. Therefore, cox proportional regression models were performed and when controlling for both the age at diagnosis and the variations in stage across tumor types, there is 2.3 times higher hazard of dying from MANEC in comparison to carcinoid and hazard is similar to GCC. Importantly, the OS for stage IV only disease of 1.5 years (95% CI 1.2–1.9) was also significantly shorter than carcinoid and more comparable to signet ring cell tumors and GCC, suggesting that, in fact, MANEC is an aggressive clinical entity. Importantly, over time as MANEC became recognized as a distinct clinical entity by the WHO in 2010 and SEER in 2007, treatment of mixed histology tumors may have evolved. Our study spans a wide time frame from 1973 to 2012, and OS for patients diagnosed after 2007 MANEC is NR (95% CI 3.7–NR) and for signet ring 2.2 years (95% CI 1.8–2.7). The majority of patients in this 6-year time frame for GCC, carcinoid, and the MANEC group are still alive, and therefore median OS is NR. This fact limits our ability to account for evolving therapies over time. Despite these limitations, these findings suggest that MANEC is a more aggressive entity that carcinoid.

These findings are consistent with our previously published experience with MANEC tumors (*n* = 46) at a single institution (22). We found that OS was 4.1 years, which was statistically significantly shorter in comparison to 13.4 years for carcinoid tumor, 15.4 years for GCC, and longer than 2.2 years for signet ring carcinoma (*p* < 0.05). The OS for stage IV appendiceal only MANEC was also statistically significantly shorter than carcinoid tumor (*p* < 0.05). Differences in OS between this study and our previous review may be related to the fact that this study includes appendiceal only tumors, and the previous data include appendiceal, small bowel, and colonic tumors. Based on that study, it is recommended that patients with MANEC undergo multidisciplinary oncologic management, which may include systemic therapy and right hemicolectomy with possible cytoreductive surgery with HIPEC in patients with peritoneal metastases.

In contrast to our findings, La Rosa et al. found that there was not a statistically significant difference in OS between MANEC and carcinoid tumors. This finding may be explained by the low sample size of 12 in their study in comparison to this larger sample size. Additionally, there were differences in the distribution of stage at diagnosis between La Rosa et al. and our study. They found 83 and 17% of patients presented with Stage III and Stage IV disease, respectively, whereas we found 16 and 30% of patients presented Stage III and Stage IV disease, respectively. Due to limitations of the SEER database, we were unable to assess immunohistochemical profiles or morphologic profiles. However, La Rosa et al. did show that 92% of MANECs had lymphatic invasion, angioinvasion, perineural invasion, consistent with the more aggressive biology (10).

Previous attempts have been made to create a separate staging system for appendiceal tumors. Turaga et al. suggested creating a staging system for appendiceal tumors where subgroup A would include patients with carcinoid and carcinoma/mucinous carcinoma and subgroup B would included those with GCC and signet ring cell carcinoma. Notably, patients in subgroup B have a three times HR in comparison to all other stages (13). Tang et al. classified appendiceal tumors with both neuroendocrine and glandular differentiation. Group A consisted of typical GCC, Group B was adenocarcinoma ex GCC, signet ring cell type, and Group C was adenocarcinoma ex GCC, poorly differentiated carcinoma type. According to the 2010 classification, Group B tumors are likely most consistent with MANEC tumors. Mean OS for Group B was 43 ± 6 months, which is shorter than our finding of 5.2 years. They also suggested that all patients in Group B and C should undergo right hemicolectomy as part of their management (23). In future classification schemas, it appears prudent to include MANEC given its recognition by WHO and its biological behavior appearing more similar to signet ring than that of GCC or carcinoid.

There are several limitations associated with this study. Although this study represents the largest analysis of MANEC patients, it is retrospective in nature, which limits definitive conclusions being drawn to determine the optimal management of MANEC patients. Additionally, the SEER database is limited in the clinicopathological characteristics assessable to investigate deeper into those variables such as therapies (systemic and surgical) associated with outcomes and survival. Additionally, the morphology was not reviewed by a single group of pathologists to ensure consistent use of terms.

Nonetheless, it appears evident from this population database and our institutional data that it is of utmost importance to distinguish MANEC from both GCC and carcinoid/neuroendocrine tumors given its more aggressive biology demonstrated. We recommend that patients with MANEC undergo aggressive multidisciplinary oncology management that may include systemic therapy and well-selected aggressive surgical management. Future prospective studies are required to more conclusively define optimal management for MANEC patients.

## Author Contributions

SB was involved in the acquisition, analysis, and interpretation of data and drafting the work. MY, TB-S, WF, JH, CS, MD, CW, and SA-M were all involved in the conception of the work and revised it critically for important intellectual content. LW was involved in the analysis of data for the work and revised it critically for important intellectual content. All authors had final approval of the version to be published and are in agreement to be accountable for all aspects of the work in ensuring that questions related to the accuracy or integrity of any part of the work are appropriately investigated and resolved.

## Conflict of Interest Statement

The authors declare that the research was conducted in the absence of any commercial or financial relationships that could be construed as a potential conflict of interest.
